# A combined 3D-SIM/SMLM approach allows centriole proteins to be localized with a precision of ∼4–5 nm

**DOI:** 10.1016/j.cub.2017.08.009

**Published:** 2017-10-09

**Authors:** Lisa Gartenmann, Alan Wainman, Maryam Qurashi, Rainer Kaufmann, Sebastian Schubert, Jordan W. Raff, Ian M. Dobbie

**Affiliations:** 1 Sir William Dunn School of Pathology, University of Oxford, Oxford, UK; 2 Micron Oxford Advanced Bioimaging Unit, Department of Biochemistry, University of Oxford, Oxford, UK; 3 Division of Structural Biology, Wellcome Trust Centre for Human Genetics, University of Oxford, Oxford, UK

## Abstract

Centrioles are small barrel-shaped structures that form centrosomes and cilia [1]. Centrioles assemble around a central cartwheel comprising the Sas-6 and Ana2/STIL proteins. The amino termini of nine Sas-6 dimers form a central hub of ∼12 nm radius from which nine dimer spokes radiate, placing the Sas-6 carboxyl termini at the outer edge of the ∼60 nm radius cartwheel [2]. Several centriole proteins are distributed in a toroid around the cartwheel, and super-resolution light microscopy studies have measured the average radii of these ∼100–200 nm radius toroids with a ‘precision’ — or standard deviation (s.d. or 1σ) — of ±∼10–40 nm. The organization of Ana2/STIL within the cartwheel, however, has not been resolvable. Here, we develop methods to calculate the average toroidal radius of centriolar proteins in the ∼20–60 nm range with a s.d. of just ±∼4–5 nm, revealing that the amino and carboxyl termini of Ana2 are located in the outer cartwheel region.

## Teaser

Centrioles are small structures that assemble around a central cartwheel comprising Sas-6, Ana2/STIL and Sas-4. The cartwheel cannot be resolved by conventional light microscopy. Here, Gartenmann et al. resolve the relative positions of the Sas-6, Ana2 and Sas-4 termini within the cartwheel using combined SIM/SMLM super-resolution microscopy.

## Main Text

**Figure 1:**
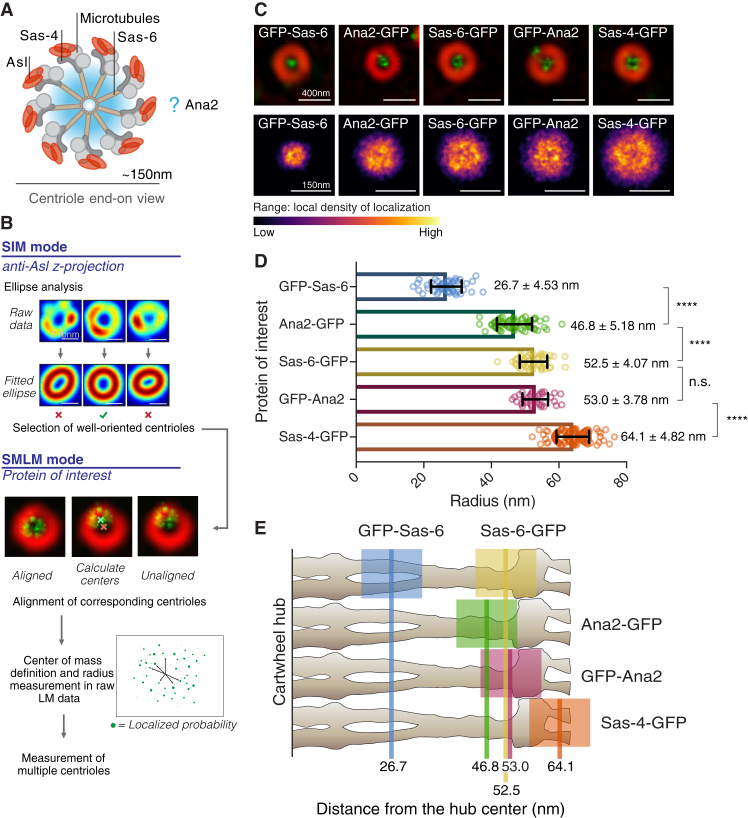
Using combined 3D-SIM/SMLM to localize centriole proteins. (A) A schematic representation of the molecular organization of a typical *Drosophila* centriole viewed from the proximal end; most centrioles in wing discs are oriented in this way relative to the apical cortex of the tissue [10]. (B) A schematic summary of the methods used to select well-oriented centrioles with SIM data, to find and align the centroids of the SIM and SMLM data, and then measure the radial distance from the SMLM data (see Supplemental Information for details). (C) The upper row of panels shows aligned 3D-SIM (*red*) and SMLM (*green*) images. The lower row of panels shows merged heatmaps of all the SMLM localizations acquired for each GFP fusion. The SMLM localizations were all normally distributed (confirmed by Gaussian fitting). (D) The bar chart illustrates the mean radii (nm) of the indicated GFP moieties within the centriole. The differences in radii are significant (unpaired T-test) for all proteins except Sas-6-GFP and GFP-Ana2 (^∗∗∗∗^ = *P*-value of 0.0001). The mean radius ± s.d. (1σ) are displayed on the right of each bar. Error bars represent s.d. (E) The average position of each GFP moiety (solid line) is shown superimposed on a schematic representation of the *Trichonympha* EM-tomogram-derived cartwheel structure [2]. The possible range of the average protein termini positions are indicated by the colored boxes. Note that the amino terminus of Sas-6 is localized too far away from the central hub; this is probably because our method is likely to slightly overestimate smaller radial distances (see Supplemental Information).

3D-structured illumination microscopy (3D-SIM) has a resolution of ∼100–160 nm, and has been used to localize several centriole proteins with average radii typically in the ∼100–200 nm range 3, 4, 5. Single-molecule localization microscopy (SMLM) can localize individual fluorescent proteins with a precision of ∼5–20 nm [6], but it is not ideal for measuring protein distribution within an organelle as only a small fraction of the total relevant proteins are usually detected. Here we have developed a novel 3D-SIM/SMLM approach to measure the radial distance of centriole proteins with a s.d. of just ∼4–5 nm (Figure 1A,B and Supplementary Information).

*Drosophila* larval wing discs expressing amino- or carboxy-terminal GFP fusions to either Sas-6 or Ana2 or a carboxy-terminal fusion to Sas-4 — a protein whose carboxy-terminal region binds to the amino-terminal region of Ana2 7, 8 — were fixed and incubated with primary and secondary antibodies to label the outer centriole protein Asl, and an Atto-488-coupled nanobody to label GFP. Asl is a component of the centriole/pericentriolar material and surrounds the mother centriole [9], ensuring that we only analyzed mother centrioles, which contain a fully assembled cartwheel. Z-projections of 3D-SIM images of Asl rings were fitted by an elliptical annular Gaussian profile, allowing us to select only well-oriented centrioles (as determined by an eccentricity of less than 1.2) for further analysis (Figure 1B). These Asl rings had a radius of ∼145 ± 5.5 nm, consistent with, and improving upon the precision of, previous measurements 3, 5 (Figure S1A).

We then collected SMLM localization data for each GFP fusion protein (Figure 1C, top panels). Linear image fitting was used to approximately align the 3D-SIM and SMLM data. Fitted centers of the 3D-SIM Asl rings were used as an initial centroid estimate for the SMLM centrioles. SMLM localizations within a 100 nm distance of this estimated centroid were selected, and a new center was determined by a weighted mean. This was repeated until the SMLM centroid positions converged. The calculated centroid positions of the 3D-SIM and SMLM datasets were aligned for image generation (Figure 1C, upper panels).

Next, a histogram of the SMLM localization radii was used to find the peak radial distance (Figure S1B). An average localization radius for each centriole was then calculated from all high-precision localizations within a distance of up to two times the peak radius (50–120 nm, depending on the protein labeled). Centrioles with a low labeling density were discarded. A weighted mean radial distance was determined. Finally, a mean-of-means was calculated to determine the average radial positions and s.d. (Figure 1D). The final number of localizations per protein was in the range of 1,200 to 8,800 from ∼40–100 centrioles (Figure S1C). Averaged centriole images from the SMLM data were created by summing all localizations that met the final criteria (Figure 1C, lower panels).

From these data we calculated the average radial distance of GFP on the Sas-6, Ana2 and Sas-4 fusion proteins with high precision (s.d. in the range of ±3.78–5.18 nm) (Figure 1D). In Figure 1E we illustrate the average relative position of each GFP (indicated by a solid line) superimposed on an outline on the EM-tomogram-derived cartwheel structure from *Trichonympha* [2]. As GFP and the anti-GFP-nanobody are a combined ∼7.5 nm in size, we show a box of ±7.5 nm around each average.

Our data confirm the relative localization of the termini of Sas-6 within the centriole cartwheel inferred from previous EM and crystallography studies [2]. They also reveal, for the first time, that both the amino and carboxyl termini of Ana2 are located in the outer cartwheel region, with the carboxyl terminus slightly closer to the hub than the amino terminus (Figure 1D). Although the average distance between the amino and carboxyl termini is small (∼6 nm), it is highly significant (*P* < 0.0001), illustrating the power of our methods. This localization is consistent with the amino terminus of Ana2 interacting with the carboxyl terminus of Sas-4 at the cartwheel periphery 7, 8. We conclude that Ana2 is likely to promote the assembly of the Sas-6 cartwheel primarily through interactions with the cartwheel spokes, rather than with the central hub. It will be important to establish whether Ana2/SAS-5/STIL proteins are similarly localized in other species.

The 3D-SIM/SMLM pipeline we develop here — using the 3D-SIM data and the weighted SMLM localization data to iteratively find the centroid of an object and then to calculate a weighted average radial distance of all the localization points — allows us to effectively combine several thousand high-precision localizations to calculate a mean localization with an s.d. of only ∼4–5 nm. We believe these methods will be broadly applicable. Our code can be easily adapted to look at other structured organelles that have an approximately circular axis, such as nuclear pores or cilia/flagella. With relatively simple modifications, this code could be used to position molecules within organelles that are highly structured, but are not circular, such as kinetochores or the long axis of chromosome arms. Finally, it should be possible to extend these methods to organelles that have an approximately regular shape, such as a mitochondrion or bacterium, to calculate the position of specific molecules relative to an ‘average’ shape.

## Author Contributions

L.G., A.W., M.Q., J.W.R. and I.M.D. designed experiments and analyzed data; L.G. performed all experiments and collected all the data; A.W. helped with sample preparation; R.K. and S.S. helped with imaging and the use of the fastSPDM; I.M.D. wrote the custom image analysis code with the help of M.Q.; L.G., J.W.R. and I.M.D. wrote the paper with the help of all other authors.

## Supplemental Information

Document S1. Experimental Procedures and One Figure

## References

[1] Nigg E.A., Raff J.W. (2009). Centrioles, centrosomes, and cilia in health and disease. Cell.

[2] Guichard P., Hachet V., Majubu N., Neves A., Demurtas D., Olieric N., Fluckiger I., Yamada A., Kihara K., Nishida Y. (2013). Native architecture of the centriole proximal region reveals features underlying its 9-fold radial symmetry. Curr. Biol..

[3] Mennella V., Keszthelyi B., McDonald K.L., Chhun B., Kan F., Rogers G.C., Huang B., Agard D.A. (2012). Subdiffraction-resolution fluorescence microscopy reveals a domain of the centrosome critical for pericentriolar material organization. Nat. Cell Biol..

[4] Lawo S., Hasegan M., Gupta G.D., Pelletier L. (2012). Subdiffraction imaging of centrosomes reveals higher-order organizational features of pericentriolar material. Nat. Cell Biol..

[5] Fu J., Glover D.M. (2012). Structured illumination of the interface between centriole and peri-centriolar material. Open Biol..

[6] Betzig E., Patterson G.H., Sougrat R., Lindwasser O.W., Olenych S., Bonifacino J.S., Davidson M.W., Lippincott-Schwartz J., Hess H.F. (2006). Imaging intracellular fluorescent proteins at nanometer resolution. Science.

[7] Cottee M.A., Muschalik N., Wong Y.L., Johnson C.M., Johnson S., Andreeva A., Oegema K., Lea S.M., Raff J.W., van Breugel M. (2013). Crystal structures of the CPAP/STIL complex reveal its role in centriole assembly and human microcephaly. eLife.

[8] Hatzopoulos G.N., Erat M.C., Cutts E., Rogala K.B., Slater L.M., Stansfeld P.J., Vakonakis I. (2013). Structural analysis of the G-box domain of the microcephaly protein CPAP suggests a role in centriole architecture. Structure.

[9] Novak Z.A., Conduit P.T., Wainman A., Raff J.W. (2014). Asterless licenses daughter centrioles to duplicate for the first time in Drosophila embryos. Curr. Biol..

[10] Roque H., Wainman A., Richens J., Kozyrska K., Franz A., Raff J.W. (2012). Drosophila Cep135/Bld10 maintains proper centriole structure but is dispensable for cartwheel formation. J. Cell Sci..

