# Supplementary material for: A combined 3D-SIM/SMLM approach allows centriole proteins to be localized with a precision of ∼4–5 nm
Source: Curr Biol. 2017 Oct 9;27(19):R1054–5. doi: 10.1016/j.cub.2017.08.009 (PMC5640508; doi:10.1016/j.cub.2017.08.009)
Supplement: Document S1. Experimental Procedures and One Figure [file mmc1.pdf]

Supplemental Information

A combined 3D-SIM/SMLM approach allows centriole proteins to be localized with a precision of ~4-5nm

Lisa Gartenmann, Alan Wainman, Maryam Qurashi, Rainer Kaufmann, Sebastian Schubert, Jordan W. Raff and Ian M. Dobbie

Figure S1

A

	Protein	Tissue	*n*	Mean radius (nm)	s.d. (nm)	ref.
SIM	anti-Asl NT	Wing discs	278	145.4	5.5	This paper
SIM	anti-Asl NT	S2 cells	21	138	15	[S1]
SIM	anti-Asl	D.Mel-2 cells		170	20	[S2]
SMLM	Asl NT	Wing discs	91	144.7	8.8	This paper

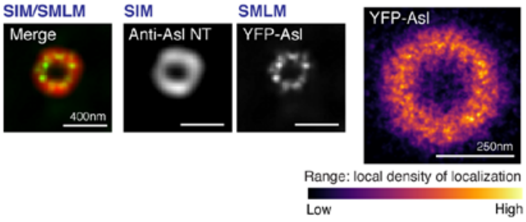

B

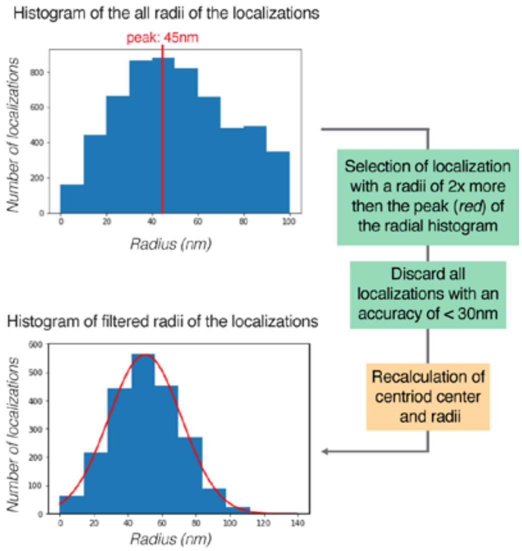

C

Protein	Centrioles selected	Centrioles measured	Total number of localisations	Number of localisations used
GFP-Sas-6	94	49	4170	1237
Ana2-GFP	136	66	11750	4744
Sas-6-GFP	102	46	11113	4091
GFP-Ana2	98	37	8525	2651
eSas-4-GFP	198	102	18332	8811
UbqSas-4-GFP	47	19	2796	1170
YFP-Asl	127	91	22023	12245

D

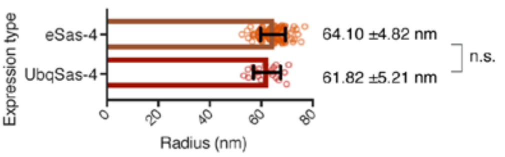

(A) Top rows compare the calculated radius of the Asl protein from our 3D-SIM measurement and previously published 3D-SIM measurements [S1,S2]. Note that the measurements were made with different antibodies in different tissues, so they would not necessarily be expected to be the same, but the s.d. of our measurements are improved by ~3-fold compared to previous measurements. Bottom row shows the calculated radii of YFP-Asl measured by SMLM. Note that the 3D-SIM and SMLM data both give a nearly identical estimate of the radius, but that the 3D-SIM data actually has a slightly smaller s.d. than the SMLM data. This is likely because the size of the Asl ring is above the resolution of the 3D-SIM system, and so the 3D-SIM — which samples all fluorophores in the Asl ring, not just the subset measured by SMLM — gives a more accurate estimation of protein distribution. Panels

below the data table show images of the aligned 3D-SIM (*red* in merged image) and SMLM (*green* in merged image) Asl data, and a merged heatmap of all the SMLM Asl localizations measured. (B) Illustration of the filtering process used to analyze the SMLM datasets, using the Ana2-GFP data set as an example. The top graph shows the initial histogram of the radii of all the Ana2-GFP SMLM localizations (5823 in total) used to calculate a peak radius. The peak radius (45nm in this case) was multiplied by 2 (giving a figure of 90nm), and any localizations that were more than 90nm away from the peak value, or had a precision less than 30nm, were discarded. These localizations were then used to recalculate the centroid position, generating the histogram shown in the bottom graph (2120 localizations in total). The SMLM data from the other fusion proteins were analyzed in a similar manner, and Gaussian fitting (*red* line) was used to confirm that these final datasets were normally distributed. (C) Table shows the number of centrioles initially selected by hand for analysis, the number of centrioles measured (that have an eccentricity of less than 1.2), the total number of SMLM localizations initially acquired for each GFP- or YFP-fusion, and the number of SMLM localizations ultimately used to calculate the radial distance of each protein. (D) Graph shows a comparison of the calculated radii of Sas-4-GFP when the fusion is expressed at either endogenous levels (eSas-4-GFP) or moderately overexpressed (UbqSas-4-GFP). The calculated radii are not significantly different (unpaired T-test; P=0.0641). Error bars represent s.d.

Supplemental Experimental Procedures

Fly lines

The following fly stocks were used in this study: eSas-4-GFP [S3], eAna2-GFP and eSas-6-GFP — all of which express the fusion proteins transgenically, but from their endogenous promoters, and are expressed at levels approximately equal to the endogenous proteins [S4] (Aydogan et al., submitted); Ubq-GFP-Ana2 [S5], Ubq-GFP-Sas-6 [S6], Ubq-Sas-4-GFP [S7] and Ubq-YFP-Asl [S8]; all of which transgenically express the fusion proteins from the Ubq promoter usually resulting in a moderate level of overexpression (~5-10 fold, depending on the tissue) [S4,S6,S9]. The moderate overexpression of the cartwheel protein GFP-fusions does not seem to alter centriole structure or size [S10], and the localization radii derived from the Ubq-Sas-4-GFP and eSas-4-GFP lines were very similar (Figure S1D). Moreover, we have previously shown that these GFP-fusion proteins can rescue the centriole duplication defects in their respective mutants, indicating that they are functional [S4,S6,S9]. Thus, the localization of these GFP-fusion proteins is likely to accurately reflect the localization of the endogenous proteins, although it is important to note that we cannot be certain that this is the case.

Larval wing disc preparation

Wing discs were dissected in PBS from 3rd instar larvae, fixed in 3.6% formaldehyde in PBS for 20min and then placed in 10ul PBS on a high precision 170 ± 5µm coverslip. The coverslip was placed on a #1 thickness coverslip and put in between two pieces of Whatman paper, and then

squashed for 10 seconds. The coverslip sandwich was then placed in liquid nitrogen for 10min. The #1 thickness coverslip was removed using a razor blade and the high precision coverslip with sample attached was placed in 100% Ethanol for 15min at -20°C, then placed in 0.1% TritonX-PBS for 10min and then washed 3x in PBS for 5min. The coverslips were incubated in primary antibody overnight at 4°C, washed 3x in PBS for 20min and then incubated in secondary antibody for 2hrs at room temperature. The coverslips were washed in PBS 3x for 10min. Finally, the coverslips were mounted in 8µl of Mowiol 4-88® mounting media on a slide. The finished slides were sealed with nail polish**.** Drosophila larval wing discs were selected for this study as the centrioles in this tissue tend to be found apically and to be oriented orthogonally relative to the apical surface. The EM structure of these centrioles has been extensively analyzed [S10,S11].

Antibodies for immunofluorescence

For all samples, the following primary antibody was used: Guinea-Pig anti-Asl [S12], GFP-booster_atto488 (ChromoTek) and Goat anti-Guinea Pig IgG Alexa Flour 594 (Invitrogen) — all at 1:500 dilution.

Optical set-up

Imaging was based on a DeltaVision OMX microscope (Applied Precision/GE Healthcare) for 3D Structured Illumination Microscopy (3D-SIM) [S13,S14] with bespoke modifications to increase the laser power density for Single Molecule Localization Microscopy (SMLM) by dSTORM [S15]. The set-up consists of a 100x/1.4 NA oil objective (UPlanSApo, Olympus), 488nm diode

laser (Sapphire 488-200, Coherent) and 592nm (F-04306-01, MPB Communications) fiber laser and EMCCD cameras (Evolve 512 Delta C, Photometrics). The cameras were run at 10 MHz. For the 3D-SIM imaging, 50ms exposures at EM gain 50 with 592nm laser light between 20 to 200W/cm^2 intensity at the sample were used. Z-stacks of 1µm at 0.125µm step size were acquired using 5 phases and 3 angles per image plane. For the SMLM imaging, 2000 or 4000 frames of 50ms exposure time with EM gain 200 were acquired under 488nm illumination (intensity approx. 25kW/cm^2 at the sample). Samples were initially imaged in 3D-SIM mode, before switching to SMLM mode on the same region of interest.

Image analysis

A previous study has used 3D-SIM and SMLM to analyze the localization of the N-terminal region of Sas-6 molecules in *Chlamydomonas* basal bodies, measuring a SMLM full-width-half-maximum (FWHM) of 41 or 52nm on the two basal bodies analyzed [S16]. In this study, we combined information from 3D-SIM and SMLM data to improve the accuracy of our measurements. We only imaged mother centrioles, as only mother centrioles organize an Asl-ring [S3,S4]. This is important as it means all the centrioles we analyzed contain a fully formed cartwheel (as, unlike in vertebrates, mother centrioles retain their cartwheel in *Drosophila*) [S17]. Several key centriole assembly proteins — such as Sas-6, Sas-4 and Ana1/Cep295 [S11,S18] (Aydogan et al., submitted) — appear to be incorporated into assembling daughter centrioles and then do not turnover in mothers; thus, it is widely believed that, once

assembled, the cartwheel does not significantly change its shape or structure in *Drosophila*.

For the position determination of the single fluorescent molecules in the SMLM images, fastSPDM was used [S19], with calibrations for our hardware setup. For the 3D-SIM images, raw data was reconstructed with softWoRx 6.1.1 (Applied Precision) using wavelength specific experimental optical transfer functions, doubling the X and Y and Z resolutions. Raw and reconstructed 3D-SIM images were checked using SIMCheck [S20] to ensure valid reconstructions and minimal artifacts.

Using custom code written in python 2 (Centroid Origin Optimising Localiser-https://github.com/MicronOxford/cool), Z-stacks of reconstructed 3D-SIM images were flattened to a single plane by maximum intensity projection. A square region approximately 0.6µm on each side centered on the manually selected 3D-SIM coordinates was cropped from the image. The position of the centriole Asl rings in the 3D-SIM images were fitted with an elliptical annular Gaussian profile, obtaining fit parameters for the center, major and minor axis, angle of ellipse and the width of the resulting Gaussian (Figure 1B). In order to minimize errors due to tilt, only centrioles near normal to the focal plane — arbitrarily chosen as those that had an eccentricity (major:minor axis ratio) of less than 1.2 — were selected for further analysis (Figure 1B). Tests without this restriction generated very similar averages but with 2-3X the standard deviation (data not shown).

As the 3D-SIM and SMLM images were acquired with different cameras, we first interpolated the SIM images to the same 20nm pixel size as the SMLM images. Then the images were aligned by performing a least square rigid body fitting (xshift, yshift, angle and scale), using SMLM positions (that were hand selected from images generated by fastSPDM) and the fitted 3D-SIM locations. This method thereby averages selection errors over all the centrioles in a whole image (128x128 pixels, usually containing multiple centrioles). Approximate positions of the equivalent, well-oriented SMLM centrioles were extracted, and these initial position estimates were refined by further rounds of centroid optimization using the raw SMLM localization positions and accuracies: from the initial position estimate, SMLM localizations within a 100nm distance (except for Asl where a distance of 300nm was used) were selected and a new center of the localizations was determined by a weighted mean centroid calculation—with the weights determined by the inverse localization precision of each localization. This new center was then used for further iterations of the above process until the centroid positions converged (typically 3-7 rounds). Using the AslNT data collected in both SIM and SMLM the average position shift during this step was ~53 nm.

Once the center position had been optimized, a histogram of the radii of the localizations from the center was examined. Only localizations within a distance of up to 2 times the peak of the radial histogram were selected (50-120nm depending on the protein labeled, and 280nm for Asl) (Figure S1B). The factor of 2 was chosen to minimize contributions from the diffuse

background signal while still ensuring we are likely to include all localizations due to specific signal from the centriole protein of interest (see paragraph below). To further improve radial distance measurements, we only included localizations with a precision great than 30nm. After these two steps, centrioles with fewer than 10 localizations that met these criteria were discarded, as the low localization number meant that the center estimates from the weighted centroid could not be relied upon. For the remaining centrioles, the selected localizations were used to calculate a weighted mean radial distance, with weights again calculated from the inverse of the localization precision. A mean-of-means combines data from multiple centrioles to determine the average radial position and standard deviation. The localization ‘precision’ was defined by the standard deviation (1σ) of the measurements. The number of localizations per protein was in the range 1200-12500 from ~40-100 centrioles (Figure S1C). Averaged centriole images (Figure 1C, bottom panels and S1A, bottom right panel) were created by taking all localizations that met the final criteria, shifting them to the weighted center of that centriole, allowing the summation of localizations from every centriole used in the final results.

Importantly, we note that this methodology is likely to inherently over-estimate the average radial distance. This is because any “non-specific” localizations (i.e. that are not due to fluorescence from the protein being measured within the centriole) are increasingly likely to be encountered the further one moves away from the centroid (as an increasing total area is being assessed for localizations as one moves further away from the centroid). Thus, any non-

specific background is not evenly distributed in radial distance, but is skewed towards more distant measurements. Moreover, the smaller the real radial distance of a protein, the more acute this problem will become. We suspect this problem may help to explain why our measurement of the GFP-Sas-6 protein radial distance is likely too large. EM and structural studies strongly suggest that the N-terminal regions of Sas-6 form a central hub that has a radial distance of ~12nm. Our measurements give a radial distance of GFP-Sas-6 of ~27nm. Even if we allow that the GFP-moiety and GFP nanobody (with a combined size of ~7.5nm) are displaced away from the central hub, this would give a radial distance of ~19.5nm, still significantly smaller than the ~27nm calculated from our methods. Further measurements on proteins with a very small radial distance will be required to determine whether this is indeed a general problem. Clearly, the higher the signal-to-background ratio, the less of a problem this error is likely to be.

Supplemental References

S1. Fu, J., and Glover, D.M. (2012). Structured illumination of the interface between centriole and peri-centriolar material. Open Biol. *2*, 120104.

S2. Mennella, V., Keszthelyi, B., McDonald, K.L., Chhun, B., Kan, F., Rogers, G.C., Huang, B., and Agard, D.A. (2012). Subdiffraction-resolution fluorescence microscopy reveals a domain of the centrosome critical for pericentriolar material organization. Nat. Cell Biol. *14*, 1159–1168.

S3. Novak, Z.A., Wainman, A., Gartenmann, L., and Raff, J.W.(2016). Cdk1 phosphorylates Drosophila Sas-4 to recruit Polo to daughter centrioles and convert them to centrosomes. Dev. Cell *37*, 545–557.

S4. Novak, Z.A., Conduit, P.T., Wainman, A., and Raff, J.W. (2014). Asterless licenses daughter centrioles to duplicate for the first time in Drosophila embryos. Curr. Biol. *24*, 1276–1282.

S5. Stevens, N.R., Dobbelaere, J., Brunk, K., Franz, A., and Raff, J.W. (2010). Drosophila Ana2 is a conserved centriole duplication factor. J. Cell Biol. *188*, 313–323.

S6. Peel, N., Stevens, N.R., Basto, R., and Raff, J.W. (2007).

Overexpressing centriole-replication proteins in vivo induces centriole overduplication and de novo formation. Curr. Biol. *17*, 834–843.
S7. Cottee, M.A., Muschalik, N., Wong, Y.L., Johnson, C.M., Johnson, S., Andreeva, A., Oegema, K., Lea, S.M., Raff, J.W. and van Bruegel, M. (2013). Crystal structures of the CPAP/STIL complex reveal its role in centriole assembly and human microcephaly. eLife *2*, e01071.
S8. Varmark, H., Llamazares, S., Rebollo, E., Lange, B., Reina, J., Schwarz, H., and Gonzalez, C. (2007). Asterless is a centriolar protein required for centrosome function and embryo development in Drosophila. Curr. Biol. *17*, 1735–1745.
S9. Cottee, M.A., Muschalik, N., Johnson, S., Leveson, J., Raff, J.W., and Lea, S.M. (2015). The homo-oligomerisation of both Sas-6 and Ana2 is required for efficient centriole assembly in flies. eLife *4*, e07236.
S10. Franz, A., Roque, H., Saurya, S., Dobbelaere, J., and Raff, J.W. (2013). CP110 exhibits novel regulatory activities during centriole assembly in Drosophila. J. Cell Biol. *203*, 785-799.
S11. Saurya, S., Roque, H., Novak, Z.A., Wainman, A., Aydogan, M.G., Volanakis, A., Sieber, B., Susano Pinto, D.M., and Raff, J.W. (2016). Drosophila Ana1 is required for centrosome assembly and centriole elongation. J. Cell Sci. *129*, 2514–2525.
S12. Roque, H., Wainman, A., Richens, J., Kozyrska, K., Franz, A., and Raff, J.W. (2012). Drosophila Cep135/Bld10 maintains proper centriole structure but is dispensable for cartwheel formation. J. Cell Sci. *125*, 5881–5886.
S13. Dobbie, I.M., King, E., Parton, R.M., Carlton, P.M., Sedat, J.W., Swedlow, J.R., and Davis, I. (2011). OMX: a new platform for multimodal, multichannel wide-field imaging. Cold Spring Harb. Protoc. *8*, 899–909.
S14. Weil, T.T., Parton, R.M., Herpers, B., Soetaert, J., Veenendaal, T., Xanthakis, D., Dobbie, I.M., Halstead, J.M., Hayashi, R., Rabouille, C., and Davis, I. (2012). Drosophila patterning is established by differential association of mRNAs with P bodies. Nat. Cell Biol. *14*, 1305–1313.
S15. Johnson, E., Seiradake, E., Jones, E.Y., Davis, I., Grünewald, K., and Kaufmann, R. (2015). Correlative in-resin super-resolution and electron microscopy using standard fluorescent proteins. Sci. Rep. *5*, 9583.
S16. Hamel, V., Guichard, P., Fournier, M., Guiet, R., Flückiger, I., Seitz, A., and Gönczy, P. (2014). Correlative multicolor 3D SIM and STORM microscopy. Biomed. Opt. Express. *5*, 3326–3336.
S17. Gonzalez, C., Tavosanis, G., and Mollinari, C. (1998). Centrosomes and microtubule organisation during Drosophila development. J. Cell Sci. *111*, 2697–2706.
S18. Conduit, P.T., Wainman, A., Novak, Z.A., Weil, T.T., and Raff, J.W. (2015). Re-examining the role of Drosophila Sas-4 in centrosome assembly using two-colour-3D-SIM FRAP. eLife *4*, e08483.
S19. Grull, F., Kirchgessner, M., Kaufmann, R., Hausmann, M., and Kebschull, U. (2011). Accelerating image analysis for localization microscopy with FPGAs. International Conference on Field

Programmable Logic and Applications (FPL), IEEE, DOI: 10.1109/FPL.2011.11.
S20. Ball, G., Demmerle, J., Kaufmann, R., Davis, I., Dobbie, I.M., and Schermelleh, L. (2015). SIMcheck: a toolbox for successful super-resolution structured illumination microscopy. Sci. Rep. *5*, 15915.
